# Supplementary material for: Sustainable production of dopamine hydrochloride from softwood lignin
Source: Nat Commun. 2023 Aug 17;14:4996. doi: 10.1038/s41467-023-40702-2 (PMC10435513; doi:10.1038/s41467-023-40702-2)
Supplement: Supplementary file 1 — Supplementary Information [file 41467_2023_40702_MOESM1_ESM.pdf]

Supplementary Information

Sustainable Production of Dopamine Hydrochloride from Softwood Lignin

Lin Dong[1,2], Yanqin Wang[2*], Yuguo Dong[1], Yin Zhang[1], Mingzhu Pan[1], Xiaohui Liu[2], Xiaoli Gu[1], Markus Antonietti[3] and Zupeng Chen[1,4*]

[1]Jiangsu Co-Innovation Center of Efficient Processing and Utilization of Forest Resources, International Innovation Center for Forest Chemicals and Materials, College of Chemical Engineering, Nanjing Forestry University, Longpan Road 159, Nanjing 210037, China.

[2]Key Laboratory for Advanced Materials and Joint International Research Laboratory of Precision Chemistry and Molecular Engineering, Feringa Nobel Prize Scientist Joint Research Center, Research Institute of Industrial Catalysis, School of Chemistry and Molecular Engineering, East China University of Science and Technology, Shanghai 200237, China.

[3]Department of Colloid Chemistry, Max-Planck Institute of Colloids and Interfaces, Research Campus Golm, Am Mühlenberg 1, Potsdam 14476, Germany.

[4]Leibniz-Institute for Catalysis, University of Rostock, Albert Einstein Street, 29a, Rostock 18059, Germany.

Corresponding author: wangyanqin@ecust.edu.cn (Y. W.); czp@njfu.edu.cn (Z. C.)

Supplementary Information

Table of Contents

Supplementary Methods......3
Materials......3
Characterizations......3
2D-HSQC NMR method for the theory monomer yield......4
Derivatization Followed by Reductive Cleavage method for the theory monomer yield......5
The qualitative and quantitative analysis of obtained dopamine hydrochloride......6
Tenfold scaling up the experiment with sole spruce lignin as substrate......8
Expectations on reducing cost......9
Supplementary Figures......10
Supplementary Figure 1......10
Supplementary Figure 2......10
Supplementary Figure 3......11
Supplementary Figure 4......12
Supplementary Figure 5......13
Supplementary Figure 6......13
Supplementary Figure 7......14
Supplementary Figure 8......14
Supplementary Figure 9......15
Supplementary Figure 10......16
Supplementary Figure 11......16
Supplementary Figure 12......17
Supplementary Figure 13......18
Supplementary Figure 14......19
Supplementary Figure 15......19
Supplementary Figure 16......20
Supplementary Figure 17......20
Supplementary Figure 18......21
Supplementary Figure 19......22
Supplementary Figure 20......23
Supplementary Tables......24
Supplementary Table 1......24
Supplementary Table 2......24
Supplementary Table 3......25
Supplementary Table 4......26
Supplementary Table 5......26
Supplementary Table 6......27
Supplementary Table 7......28
Supplementary Table 8......29
Supplementary Table 9......30
Supplementary References......31

Supplementary Methods

Materials

The chemicals were purchased from commercial suppliers and used as provided: 2-(4-hydroxy-3-methoxyphenyl)-1,3-dioxolane (**1**, 98.0%, Macklin), 2-(4-hydroxy-3-methoxyphenyl)-ethanol (**2**, 97.0%, Aladdin), 2-(4-hydroxy-3-methoxyphenyl)-aldehyde (**1b**, 95.0%, Bide), 2-(4-hydroxy-3-methoxyphenyl)-ethamine (**3**, 95.0%, Macklin), 2-(4-hydroxy-3-methoxyphenyl)-propanol (**1a**, 98.0%, Macklin), 4-propyl-2-methoxyphenol (**1c**, 98%, Aladdin), dopamine (98.0%, Macklin), dopamine hydrobromide (**4a**, 98.0%, Aladdin), dopamine hydrochloride (**4**, 98.0%, Aladdin), methanol (99.8%, Sinopharm Chemical), ethanol (99.8%, Sinopharm Chemical), isopropanol (99.7%, Sinopharm Chemical), ethylenediaminetetraacetic acid disodium salt solution (0.1 mol/L, Macklin), leucocrystal violet (5 g/L, Regal), perchloric acid titrant (0.1 mol/L, Reagecon)1,4-dioxane (99.5%, Sinopharm Chemical), toluene (99.5%, Sinopharm Chemical), dimethyl carbonate (98.0%, Aladdin), ethylene glycol (98.0%, Aladdin), NaOH (96.0%, Sinopharm Chemical), sodium dodecyl sulfate (99.9%, Macklin), acetonitrile (99.9%, Sinopharm Chemical), glacial acetic acid (99.9%, Macklin), nitrobenzene (99.0%, Sinopharm Chemical), HCl (36.0%-38.0%, Sinopharm Chemical), H_2SO_4 (95.0-98.0%, Sinopharm Chemical), HNO_3 (65.0-68.0%, Sinopharm Chemical), HBr (40.0%, Aladdin), HI (55.0-58.0%, Aladdin), HF (40.0%, Aladdin), trifluoromethanesulfonic acid (TfOH, 98.0%, Macklin), $RuCl_3{\cdot}3H_2O$ (99.6%, Macklin), $Ni(NO_3)_2$ $6H_2O$ (98.0%, Aladdin), $FeCl_3{\cdot}6H_2O$ (99.7%, Macklin), $NH_3{\cdot}H_2O$ (99.8%, Sinopharm Chemical), TiO_2 (Aladdin), Al_2O_3 (Aladdin), CeO_2 (Macklin), active carbon (Rhawn), Raney Ni (Aladdin). All chemicals were used as received without further purification. Spruce wood, pine wood, cedar wood, and douglas fir wood were purchased from a local manufactory (*ca*. 40 mesh) and dried at 100 °C for 5 h before use.

Characterizations

Transmission electron microscopy (TEM) was examined by using a JEOL JEM-2100EX microscope, and the electron-beam accelerating voltage was 200 kV. The powder X-ray diffraction (XRD) patterns were recorded on a Rigaku D/max-2550VB/PC diffractometer with Cu Kα radiation (λ = 1.5406 Å). Each sample was scanned from 2θ = 10º to 80º. The analysis of Ni content in samples was performed by using inductively coupled plasma atomic emission

spectrometry (ICP-AES, Agilent 725ES ICP-AES). The analysis of Cl and Br content in samples was performed by using ion chromatography (IC, DIONEX AQUION). IC analysis was performed using a Dionex IonPac AS19 column (4×250 mm) with ampere detector. The mobile phase was a KOH aqueous solution (20 mmol/L) at a flow rate of 1.0 $mL\cdot min^{-1}$ and the column was kept at a temperature of 30 °C. Nitrogen adsorption/desorption isotherms were measured on a Micromeritics ASAP 2020M sorption analyzer. Before the measurements, all samples were outgassed at 180 °C for 12 h under vacuum to remove moisture and impurities from pores. The surface area was calculated by the Brunauer-Emmett-Teller (BET) method, the pore size distribution was calculated by the Barrett-Joyner-Halenda (BJH) method through the desorption branch of the isotherm and the total pore volume was estimated at a relative pressure of 0.975. Mass (MS) spectra were recorded using a SCIEX X500R LC-Q-TOF, ESI ion Source. Ultraviolet-visible (UV-Vis) spectra were recorded using a Perkin-Elmer Lambda 950. Fourier transform infrared (FT-IR) spectra were recorded on a Bruker VERTEX 80 V instrument. NMR spectra were recorded on Bruker Avance-400 instrument, using D_2O as solvent. ^1H NMR spectral data were reported in terms of chemical shift (δ, ppm), multiplicity, coupling constant (Hz), and integration. ^{13}C NMR spectral data were reported in terms of chemical shift (δ, ppm). The following abbreviations indicated the multiplicities: s, singlet; d, doublet; t, triplet; q, quartet; m, multiplet; br, broad. The 2D-HSQC-NMR spectra were recorded on Bruker Avance-600 instrument. The test program follows: 10.3 ppm sweep width in the F2 (^1H), 165 ppm sweep width F1 (^{13}C), an acquisition time of 130 ms, a relaxation delay time of 1.5 s, 24 scans and 512 data points. MestReNova software was used to process the HSQC data and quantitatively analyze the composition of the sample.

2D-HSQC NMR method for the theory monomer yield

The 2D-HSQC NMR method was carried out to calculate the theory monomer yield according to early reports[1,2]. The theoretical monomer yield of lignin acid-catalyzed depolymerization is dependent on the *β*-O-4 linkages content of the lignin. As shown in Supplementary Fig. 3, the relative ratios of the main linkages of spruce lignin were determined by 2D-HSQC NMR techniques. Relative quantification of the main linkages provided a *β*-O-4/*β*-5/*β*-*β* ratio of 0.68/0.28/0.04 (Supplementary Table 1). The H (*p*-hydoxyphenyl)/G (guaiacyl)/S (syringyl) subunit ratio of 0/0.98/0.02, which corresponded well to the H/G/S

ratios of product mixtures obtained after lignin depolymerization. In addition, the *β*-O-4 to monomer ratio was 1/3.0. Based on these data, the theoretical maximum monomer yield from spruce lignin was estimated to be about 11 wt.% assuming that only the *β*-O-4 linkages were cleaved and monomeric products were only obtained when two *β*-O-4 linkages flank a monomer. The theoretical monomer yield of other softwood lignin was also calculated by the same process. The 2D-HSQC NMR characterization of pine lignin, cedar lignin, and douglas fir lignin was conducted and the partial 2D-HSQC NMR spectra were shown in Supplementary Fig. 4. The *β*-O-4 to monomer ratio of pine lignin, cedar lignin, and douglas fir lignin was 1/3.3, 1/4.1, and 1/3.4, respectively. Thus, the theoretical maximum monomer yield from pine lignin, cedar lignin, and douglas fir lignin was estimated to be about 9, 6, and 9 wt.%, respectively.

Derivatization Followed by Reductive Cleavage (DFRC) method for the theory monomer yield

The DFRC method was also carried out to calculate the theory monomer yield according to early reports[3,4]. In a 10 mL round bottom, 20 mg of lignin and 3 mL of acetyl bromide solution (20/80 AcBr/Acetic acid) were added. Then, the mixture was stirred at 130 rpm, 50 °C for 3 hours. The solvent was removed by rotary evaporation at 40 °C and the residue was dissolved in 3 mL stock solution (5/4/1 dioxane/acetic acid/ water). 50 mg zinc dust was added to a solution and stirred for 1 h. The mixture was quantitatively transferred to a separating funnel. Then, 10 mL of DCM, 10 mL of saturated NH_4Cl, and 0.2 mg of tetracosane were added. The pH of the aqueous phase was adjusted to < 3 by adding 3% HCl aqueous solution. The water phase was extracted with 5 mL DCM. Then, the combined DCM fractions were dried over $MgSO_4$ and the filtrate was evaporated under reduced pressure. The residue was further dissolved in 1.1 mL DCM and 0.4 mL stock solution (1/1 dry pyridine/acetic anhydride) was added under nitrogen. Then, the solution was vortexed and stirred for 1 h. Finally, all volatiles were co-evaporated with ethyl alcohol and the residue was dissolved DCM for GC-FID analysis. The liquid phase was analyzed by a Shimadzu GC with a HP-5 column (30 m × 0.25 μm) and run with a temperature profile: 140 °C (held for 1 min), raised at 3 °C/min to 240°C (held for 1 min), raised at 30 °C/min to 300 °C (held for 12 min). Theoretical monomer (**1**) yield was calculated using response factors with tetracosane as the internal standard (IS). The calculations were done as follows:

$m_{Gc\ or\ Gt}$ = response factor × m_{IS} / Area $_{Gc\ or\ Gt}$ (eq. 1)

mol_{Gc+Gt} = (m_{Gc} + m_{Gt}) / 264.277 (eq. 2)

The theoretical yield of **1** (wt.%) = mol_{Gc+Gt} × 210.23 / m_{lignin} (eq. 3)

Depolymerization efficiency (%) = yield of **1** / theoretical yield of **1** (eq.4)

The GC-FID chromatogram of DFRC monomers from spruce lignin and the mass spectrogram of G-monomers were shown in Supplementary Fig. 8. The theoretical monomer yield of spruce lignin is 10.8 wt.%, which is consistent with the results from 2D-HSQC NMR method (~11 wt.%). After the acid-catalytic depolymerization of spruce lignin using ethylene glycol as a stabilization agent, the yield of **1** was up to 10.3 wt.% with high depolymerization efficiency (95.4%). The quantitative analysis of the theoretical monomer yield of other softwood lignin was conducted in the same method. The theoretical yield of pine lignin, cedar lignin, and douglas fir lignin was 8.8, 5.4, and 9.5 wt.%, consistent with the 2D-HSQC NMR results (~9, 6, and 9 wt.%). Combined with the yield of **1**, the depolymerization efficiency of pine lignin, cedar lignin, and douglas fir lignin was 97.7%, 96.3%, and 83.2%, respectively (Table 1).

The qualitative and quantitative analysis of obtained dopamine hydrochloride

According to Chinese Pharmacopoeia, the obtained white powder in ethanol was qualitatively and quantitatively analyzed *via* a series of methods (https://db.ouryao.com/yd2020/view.php?id=fc6dfd4025), including the color test method, ultraviolet-visible spectroscopy (UV-Vis), Fourier transform infrared spectroscopy (FT-IR), mass spectrometry (MS), nuclear magnetic resonance (NMR), high-performance liquid chromatography (HPLC), ion chromatography (IC), and titrimetric analysis method.

a) Color test method: The white powder (10 mg) was first dissolved in deionized water (1 mL) forming a colorless solution, which changed to dark green after dripping $FeCl_3$ solution. The color of the solution was further changed to purplish red after adding$NH_3{\cdot}H_2O$ (Supplementary Fig. 20a).

b) UV-Vis method: The white powder (3 mg) was first dissolved in H_2SO_4 solution (0.5 wt.%, 100 mL) and was further measured by UV-Vis spectroscopy. As shown in Supplementary Fig. 20b, a single peak at 280 nm assigned to the benzene ring group was observed, agreeing well with the commercial dopamine hydrochloride standard.

c) FT-IR method: The white powder (1 mg) was mixed with dried KBr (0.2 g) and finely ground in an agate mortar, which was further pressed into a transparent slice for FT-IR analysis.

As shown in Supplementary Fig. 20c, the white powder presented characteristic bands at 1286, 1499, 1612, 2953, 3041, 3216-3343 cm^{-1} assigning to the stretching of C-O, C-C (aromatic), C-N, C-H (alkyl), C-H (aromatic), and -OH groups, respectively. The FT-IR spectrum of white powder was nearly the same as that of the commercial dopamine hydrochloride, suggesting that the white powder should be dopamine hydrochloride.

d) MS method: As shown in Supplementary Fig. 20d, the mass spectrum of the white powder was consistent with that of the commercial dopamine hydrochloride, further proving the structure of white powder. MS (ESI): 154 $[M+H]^+$.

e) NMR method: ^1H and ^{13}C NMR spectra for the obtained white powder were shown in Fig. 4d, which were consistent with the results of the previous studies[5]. ^1H NMR (400 MHz, D_2O): δ 6.86 (d, J = 8.0 Hz, 1H), 6.81 (s, 1H), 6.72 (d, J = 8.0 Hz, 1H), 3.19 (t, J = 7.0 Hz, 2H), 2.84 (t, J = 7.0 Hz, 2H). ^{13}C NMR (101 MHz, D_2O): δ 144.1, 142.9, 129.2, 121.1, 116.5, 116.4, 40.6, 31.9.

f) HPLC method: In most of the previous literature[6-8], the yield of dopamine hydrochloride was determined by HPLC analysis. Therefore, the yield and purity of the white powder were also determined by HPLC (Agilent 1200 Series), equipped with an Agilent C18 column (Zorbax SB-C18; 4.6 mm × 150 mm, 3.5 μm) and a diode-array detector. The mobile phase was sodium dodecyl sulfate (0.005 mol/L)/acetonitrile/glacial acetic acid/ethylenediaminetetraacetic acid disodium salt solution (0.1 mol/L) = 700/300/10/2. In brief, the obtained white powder was dissolved in the mobile phase (0.3 g/L). 10 μL of the sample solution was injected under the following conditions: column temperature = 30 °C, flow rate = 1.0 mL min^{-1}, and wavelength = 280 nm. As shown in Supplementary Fig. 20e, a peak at 10.9 min was observed in the corresponding HPLC chromatograph, consisting with the commercial dopamine hydrochloride. The yield of dopamine hydrochloride was calculated to be 92.6% using 4-ethylpyrocatechol as the internal standard. Further combined with ion chromatography (IC) results, the purity of obtained dopamine hydrochloride was determined to be 98.0 % and the major impurities were 4-(2-aminoethyl)-2-methoxyphenol hydrochloride (0.9%) and dopamine hydrobromide (0.8%).

g) Titrimetric analysis method: The obtained white powder (150 mg) was first mixed with glacial acetic acid (25 mL). After boiling at 120 ℃ for 5 mins, the solution was cooled down to 40 ℃. Next, mercury acetate test solution (5 mL) and a drop of leucocrystal violet were added to the solution. Finally, the solution was titrated with perchloric acid titrant (0.1 mol/L) until the color changed to green and corrected the result with a blank test. 1 mL of perchloric acid titrant (0.1 mol/L) is equivalent to 18.96 mg of dopamine hydrochloride. Based on titrimetric analysis, the purity of obtained dopamine hydrochloride was 98.2%, similar to

results from the HPLC method (98.0%).

Tenfold scaling-up experiment with sole spruce lignin as substrate

Firstly, the depolymerization of lignin into **1** was conducted in a 250 mL stainless-steel autoclave reactor. Spruce lignin (2.0 g) was mixed with ethylene glycol (7.2 mL), H_2SO_4 (160 μL), 1,4-dioxane (300 mL) and reacted at 140 °C for 10 h. After the reaction, the reactor was quenched in an ice-water bath and the mixtures were quantitatively analyzed by GC-FID. The yield of **1** was 9.5 wt.% based on the lignin. In this step, the obtained **1** did not need to be separated. Next, Ru/C (1.0 g) and H_2O (10 mL) were filled into the autoclave, which was sealed and charged to an initial pressure of 1.0 MPa with H_2/N_2 (50 vol%). The autoclave was heated to 120 °C for 10 h. After the deprotection reaction, the mixtures were analyzed by GC-FID and the yield of **2** was up to 6.3 wt.%. The mixtures were centrifuged to separate the liquid phase and Ru/C catalyst, and the latter was recycled for the deprotection reaction. Reduced pressure distillation of the liquid phase was carried out to achieve 1,4-dioxane recovery, which was used for lignin depolymerization again. To the resulting mixture, dichloromethane (10 mL) and saturated Na_2CO_3 solution in H_2O (20 mL) were added. The aqueous layer was extracted with dichloromethane (20 mL × 3), and the combined organic layers were dried over anhydrous Na_2SO_4, concentrated in vacuo, and subjected to silica gel flash chromatography (petroleum ether : ethyl acetate = 2:1) to afford the desired **2**. Then, the amination of **2** was tested in a fixed-bed reactor. The 10%Ni/TiO_2 catalyst packed into the tubular reactor was *in-situ* reduced at 400 °C for 4 h. A feed of **2** in *p*-xylene was injected into the fixed-bed reactor with a 0.163 h^{-1} of weight-hourly space velocity (WHSV). The reactor was heated to 160 °C with an NH_3 flow rate of 100 mL/min. After the hydrogen-borrowing amination reaction, obtained **3** was up to 5.1 wt.%. Reduced pressure distillation of the liquid phase was carried out and the recycled *p*-xylene solvent was used for the amination reaction. The crude products were concentrated in vacuo and further purified by column chromatography (dichloromethane : methanol = 100:1) leading to highly purified **3**. The **3** was mixed with HBr and reacted at 120 °C for 6 h in an oil bath under an N_2 atmosphere. After the hydrolysis reaction, the mixtures were centrifuged to separate liquid phase and solid insoluble precipitate, and the former was recycled for the hydrolysis reaction. The **4a** solid was obtained simply by drying at 50 °C in a vacuum oven. The obtained **4a** was mixed with ethanol and heated at 80 °C in an oil bath under N_2 atmosphere until fully dissolved. Then, HCl was slowly dripped into the solution and the reactor was cooled in an ice-water bath. Finally, the **4** was obtained simply by filtration and drying. The liquid phase was recycled for the conversion of **4a**. From HPLC results, the mass yield of **4** was 3.3 wt.% based on spruce lignin. In addition, the purity of **4** from the tandem conversions of lignin was 94.7%. The impurities in products were also qualitatively and quantitatively analyzed,

including 4-(2-aminoethyl) phenol hydrochloride (3.1%), 4-(2-aminoethyl)-2-methoxyphenol hydrochloride (1.1%), and **4a** (0.9%). No dimers or oligomers were detected, which were removed in the previous step *via* column chromatography method. The relatively low yield of **4** might be due to mass loss in the process of transfer, separation, and purification. We are still working on the improvement of the overall efficiency including product purification, solvent recovery, catalyst, and additive separation and recovery.

Expectations on reducing cost

The production cost of dopamine hydrochloride in this work was recalculated to be 2.20 million CNY/t (Chinese Yuan per ton), which was much lower than the dopamine hydrochloride market prices in the range of 4-6 million CNY/t over the last year[9]. Based on the results of the lignin-to-dopamine hydrochloride route and techno-economic analysis, we highlighted the following aspects to further reduce the cost for future consideration.

a) It is crucial to take into consideration of the costs of solvents, especially for large-scale applications in order to make the biorefineries be feasible economically. The substrate concentration was not high in this work, which require additional cost of solvents and energy for product separation. Thus, reaction process conditions should be further optimized to increase the concentration of substrates.

b) Due to the use of homogeneous acids (*e.g.* H_2SO_4, HBr, and HCl), the used equipment must have excellent corrosion resistance. Developing catalytic systems with heterogeneous solid acid catalysts instead of homogeneous acids is highly desired to reduce equipment costs. In addition, the heterogeneous solid acid catalysts are easy to be separated and recycled compared to the homogeneous ones.

c) In our processes, acid-catalyzed lignin depolymerization proceeds via cleavage of the β-O-4 bond to afford low-molecular-weight monomers, but the other interunit C-O linkages (*e.g.* 4-O-5 and α-O-4) and C-C linkages are not cleaved. Because of the presence of stable interunit C-O and C-C bonds within native lignin, the yield of dopamine hydrochloride from the tandem conversions of lignin is seriously limited. Designing innovative and new strategies that can conduct cleavage of both interunit C-O and C-C linkages is believed to mitigate the limitations on dopamine hydrochloride production.

d) After lignin depolymerization, the desired **1** was about 10 wt.% based on lignin and the remaining by-products are mainly phenolic oligomers. The residual phenolic oligomers can be further converted into highly valuable chemicals to achieve a high mass and carbon efficiency, such as printing ink, fuels, polymeric materials, and so on[10-12]. Maximal valorization of lignin into value products is key in demonstrating the potential of wood biorefineries.

Supplementary Figures

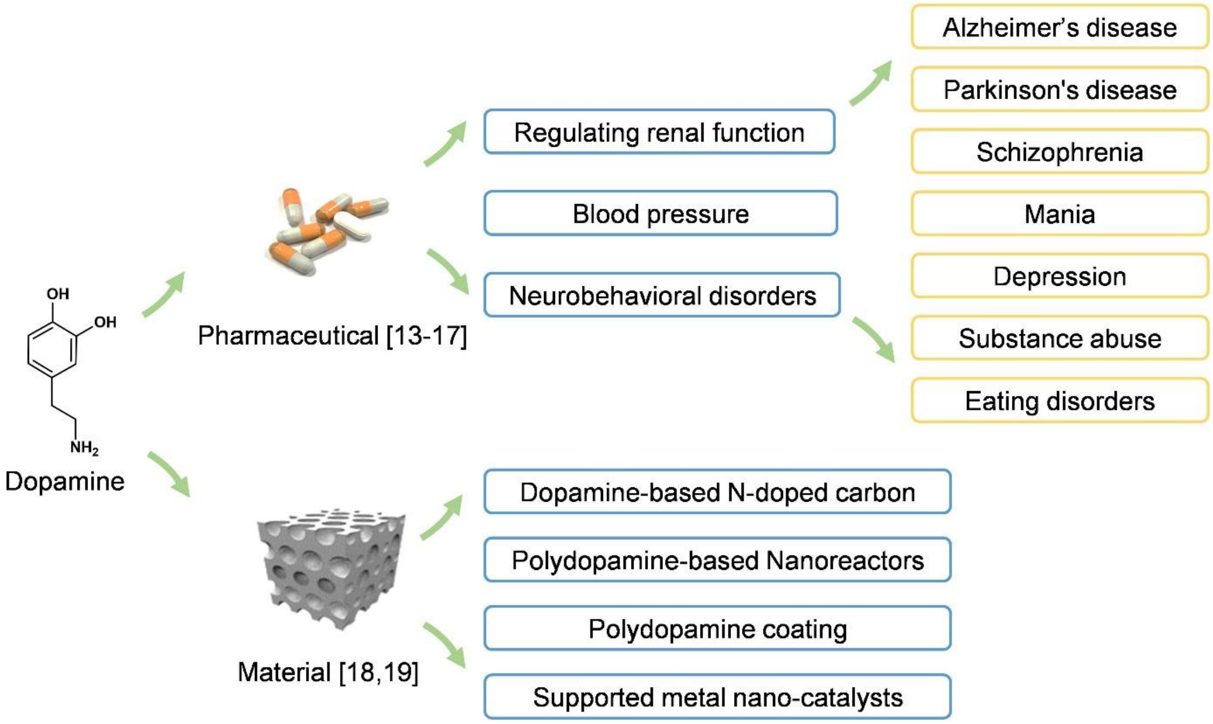

Supplementary Figure 1. The diverse application pattern of dopamine in pharmaceutical and material synthesis. The citations in the figure are listed in References.

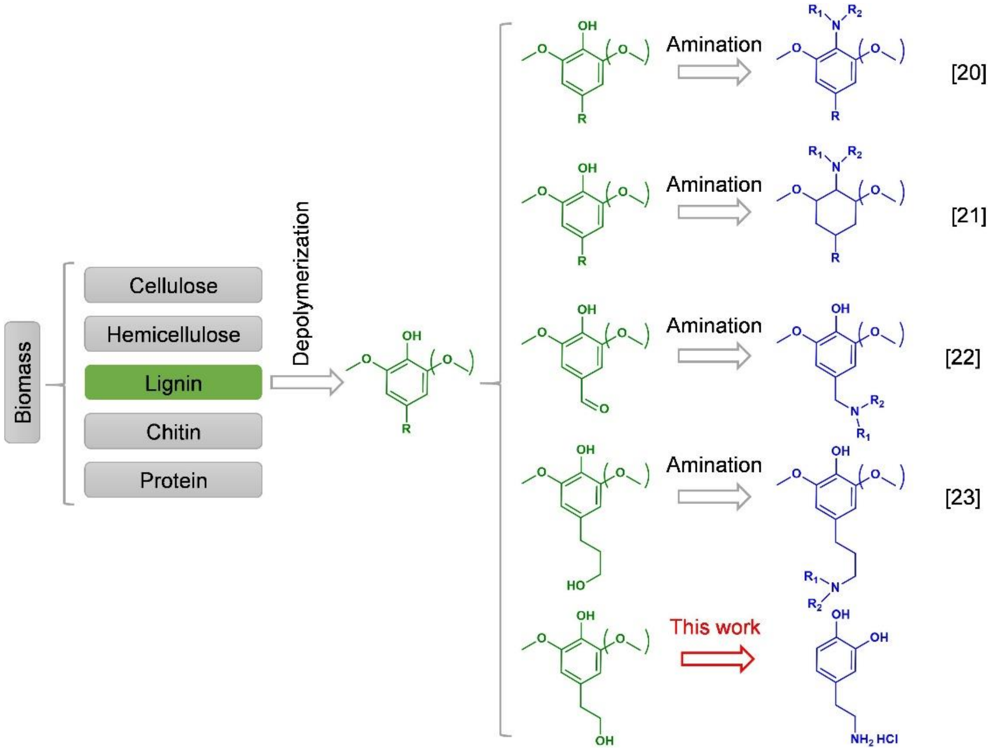

Supplementary Figure 2. Schematic representation of the conversion of lignin towards bio-based amines based on previous literature. The citations in the figure are listed in References.

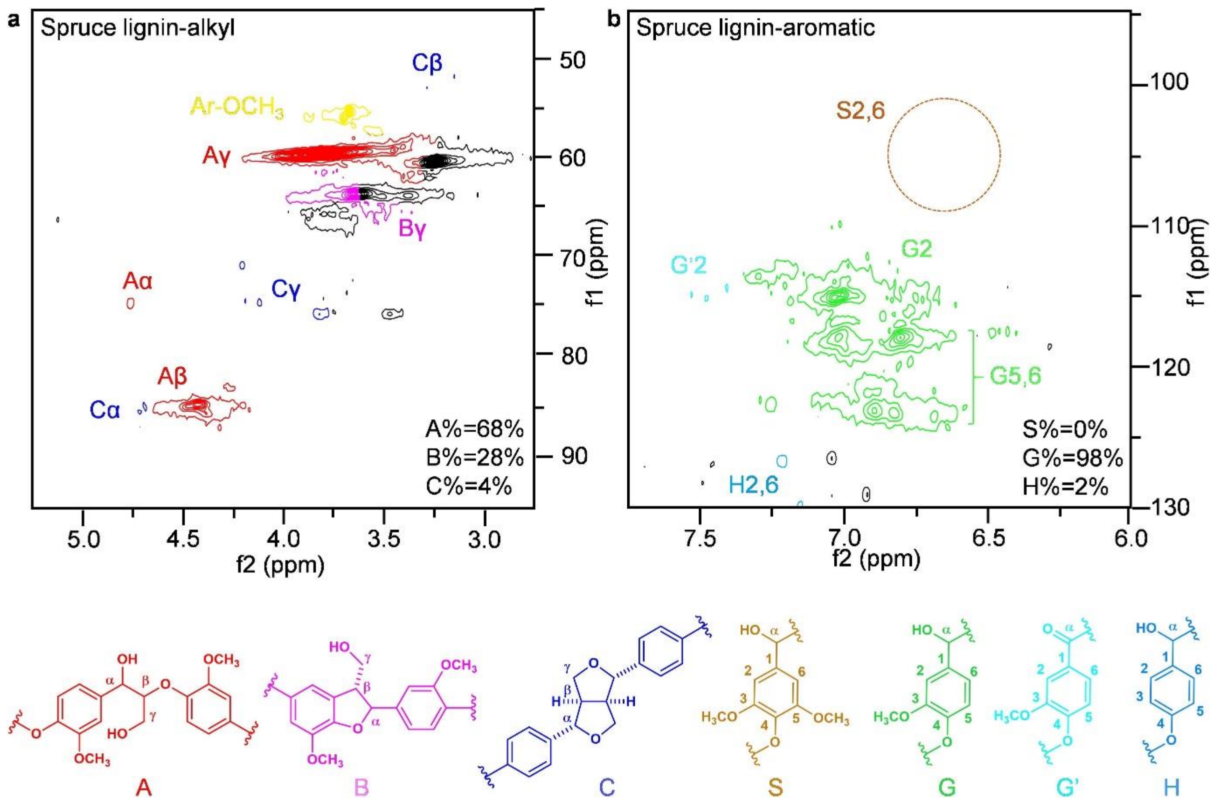

Supplementary Figure 3. (a) The alkyl region and (b) the aromatic region of the 2D-HSQC NMR spectra of spruce lignin. Some representative structures are shown underneath.

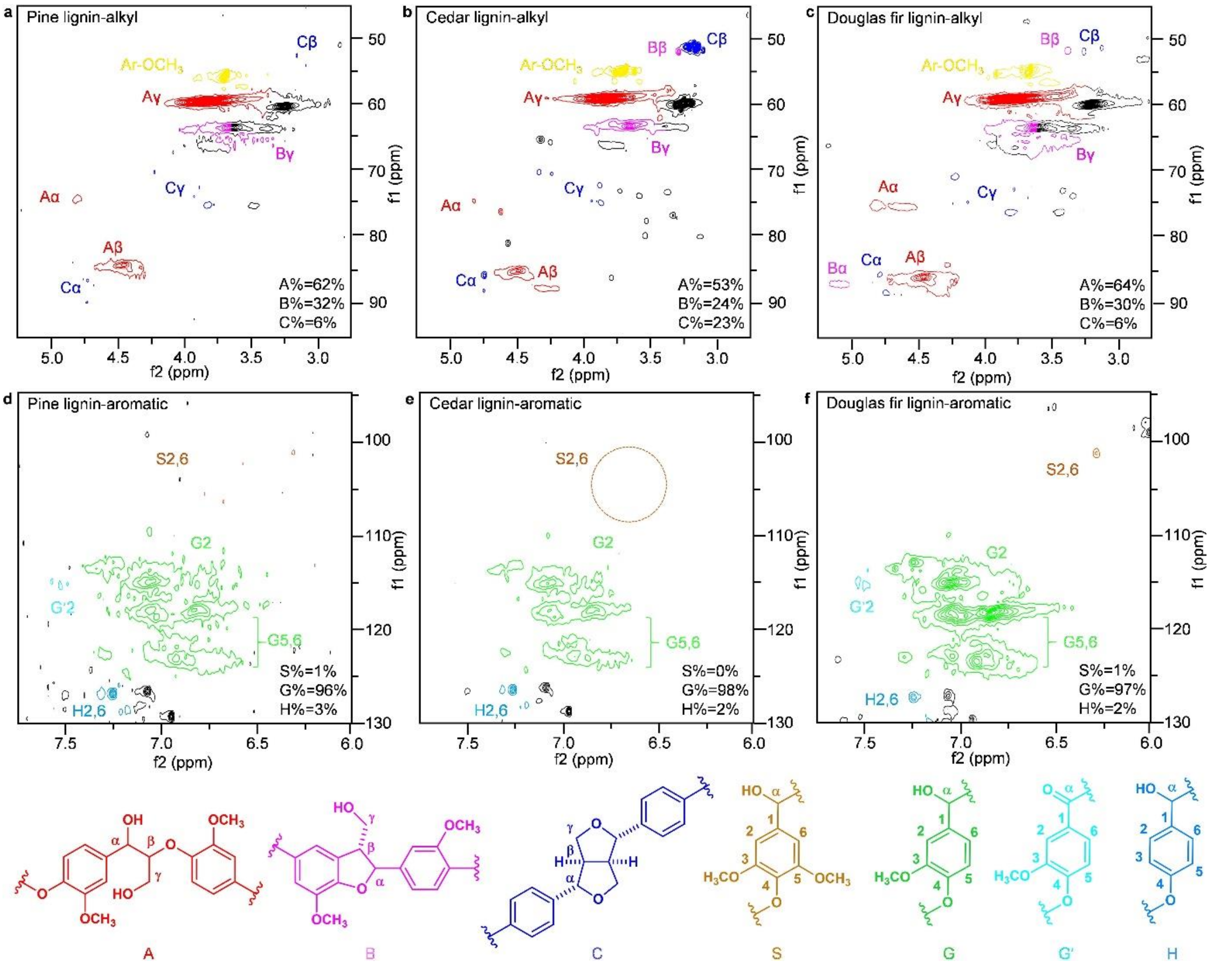

Supplementary Figure 4. (a-c) The alkyl region and (d-f) the aromatic region of the 2D-HSQC NMR spectra of pine lignin, cedar lignin, and douglas fir lignin. Some representative structures are shown underneath.

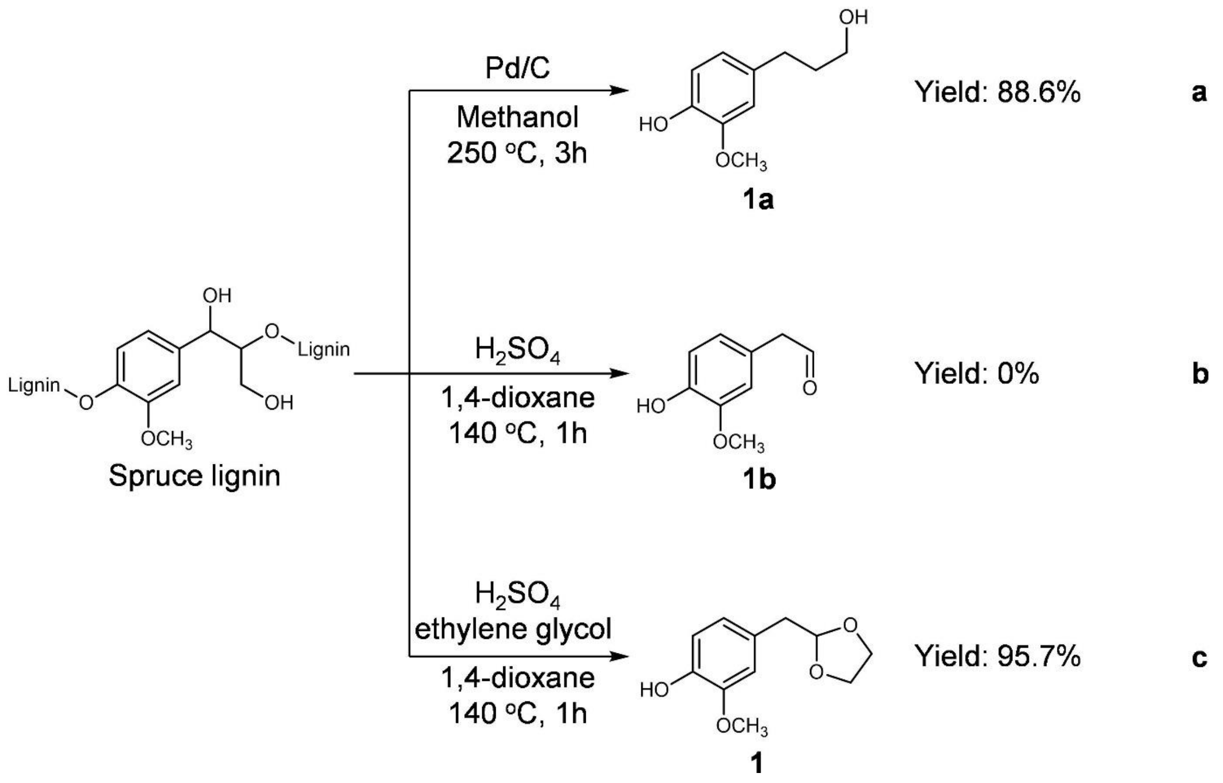

Supplementary Figure 5. Catalytic depolymerization of lignin into monolignols. Catalytic results for the depolymerization of spruce lignin with various methods in a batch reactor. Reaction conditions: **a** lignin (0.2 g), methanol (20 mL), 5%Pd/C (0.1g), H_2 pressure (3.0 MPa), temperature (250 °C), 3 h. **b** lignin (0.2 g), 1,4-dioxane (30 mL), H_2SO_4 (16 μl), N_2 pressure (1.0 MPa), temperature (140 °C), 1 h. **c** lignin (0.2 g), 1,4-dioxane (30 mL), H_2SO_4 (16 μl), ethylene glycol (0.72 mL), N_2 pressure (1.0 MPa), temperature (140 °C), 1 h.

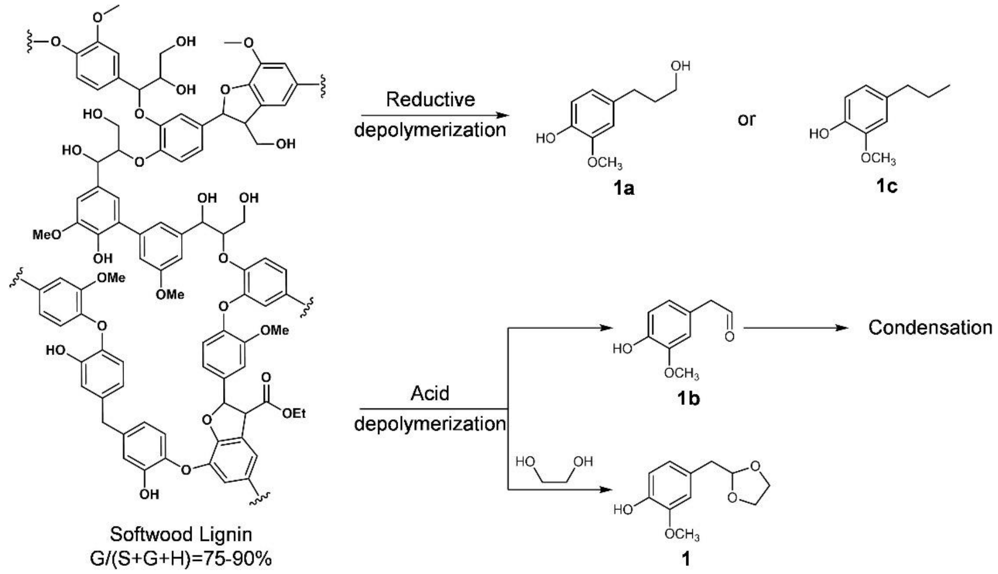

Supplementary Figure 6. Schematic representation of depolymerization of softwood lignin into monolignols. S, G and H represent syringyl, guaiacyl, and *p*-hydroxyphenyl, respectively.

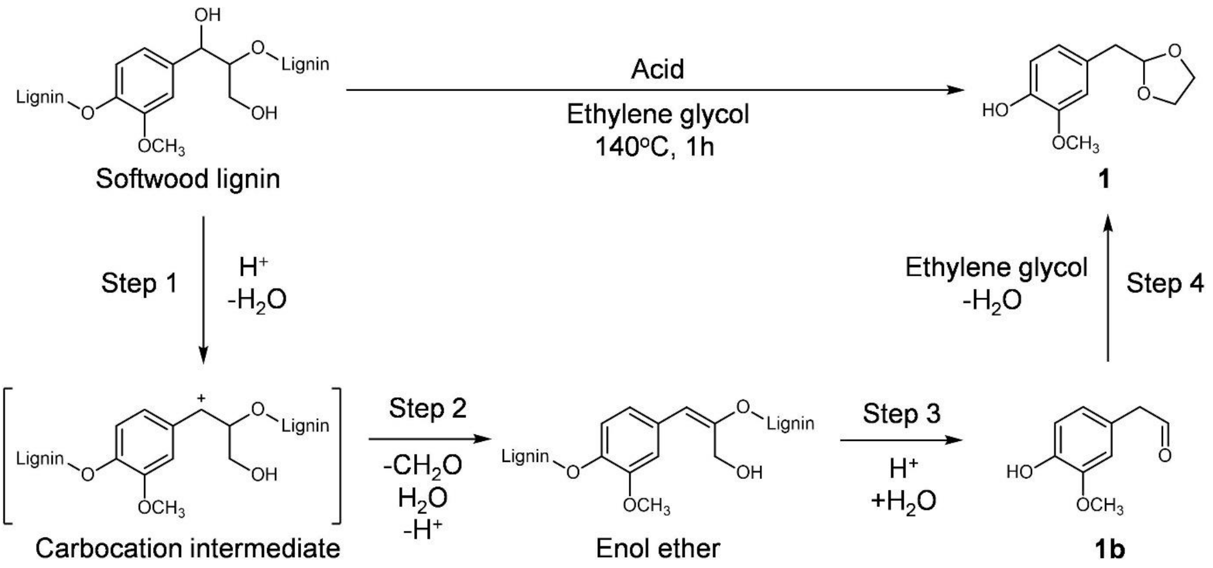

Supplementary Figure 7. Reaction mechanism for the acidolysis of softwood lignin with ethylene glycol to obtain 1.

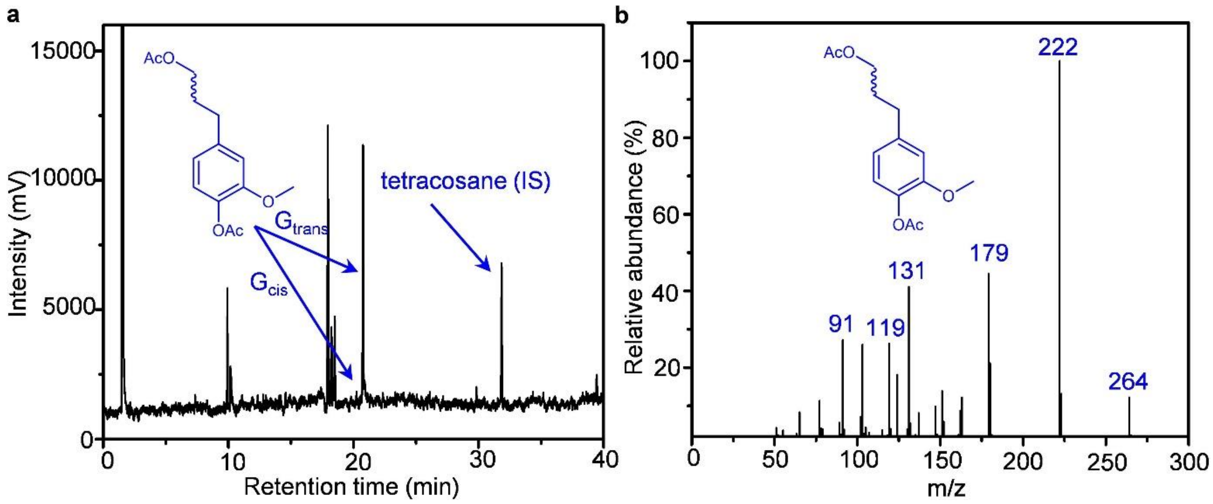

Supplementary Figure 8 (a) GC-FID chromatogram of DFRC monomers form spruce lignin and (b) the mass spectrogram of G-monomers.

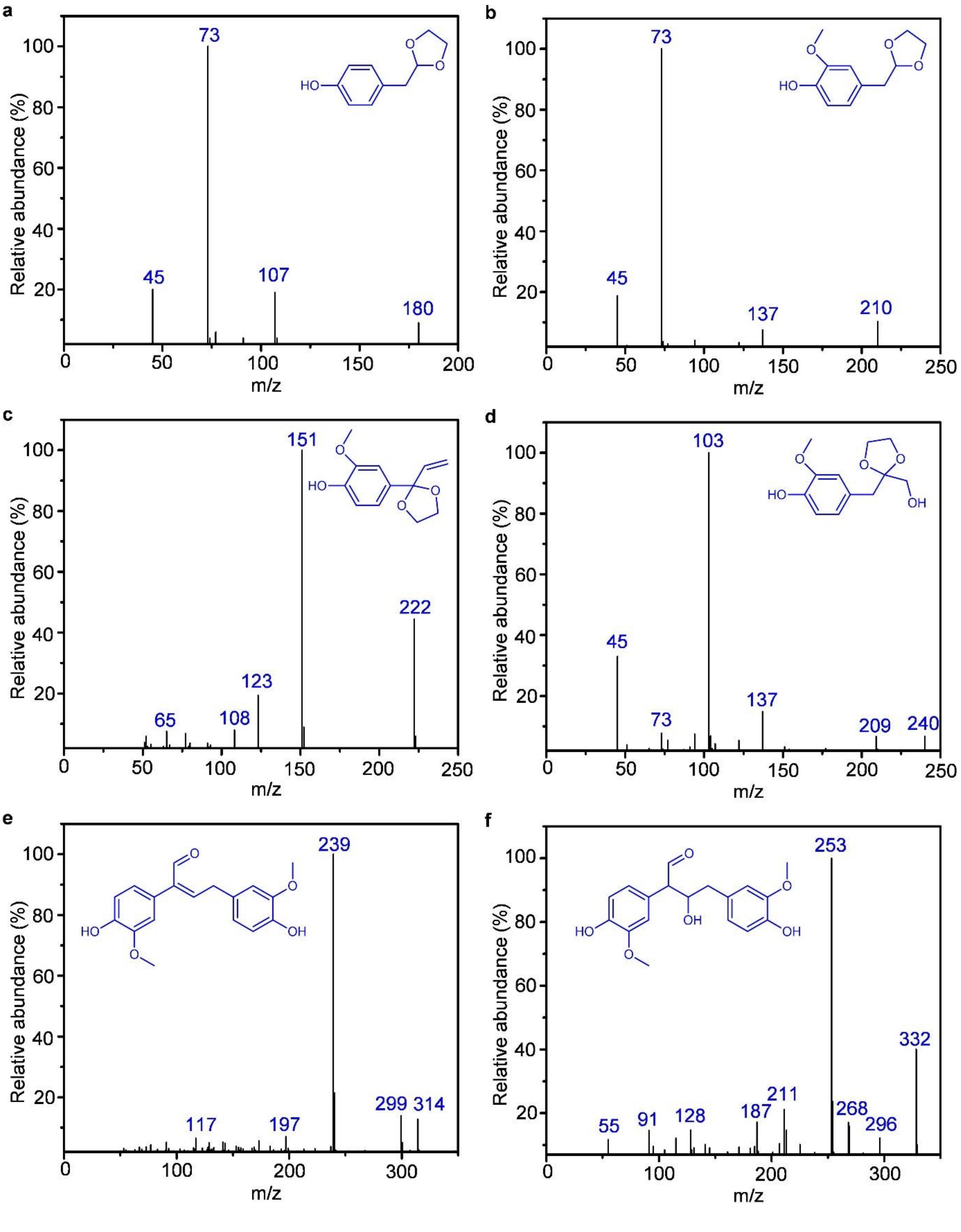

Supplementary Figure 9. GC-MS spectra of the products (entries 4, 5, 6, 7, 9, and 10) in Supplementary Table 3.

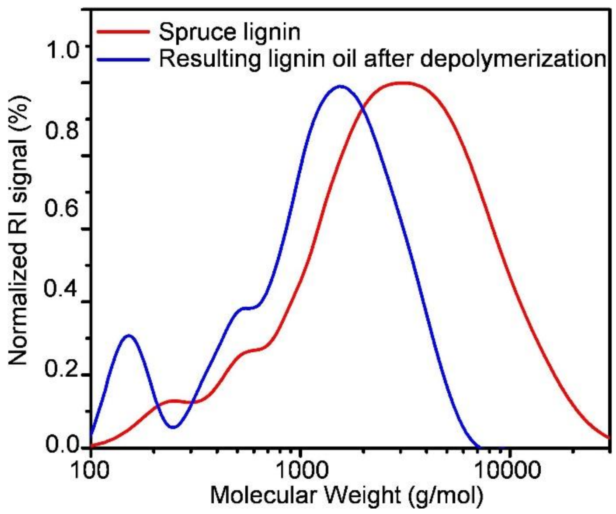

Supplementary Figure 10. GPC of raw spruce lignin and the resulting lignin oil after depolymerization.

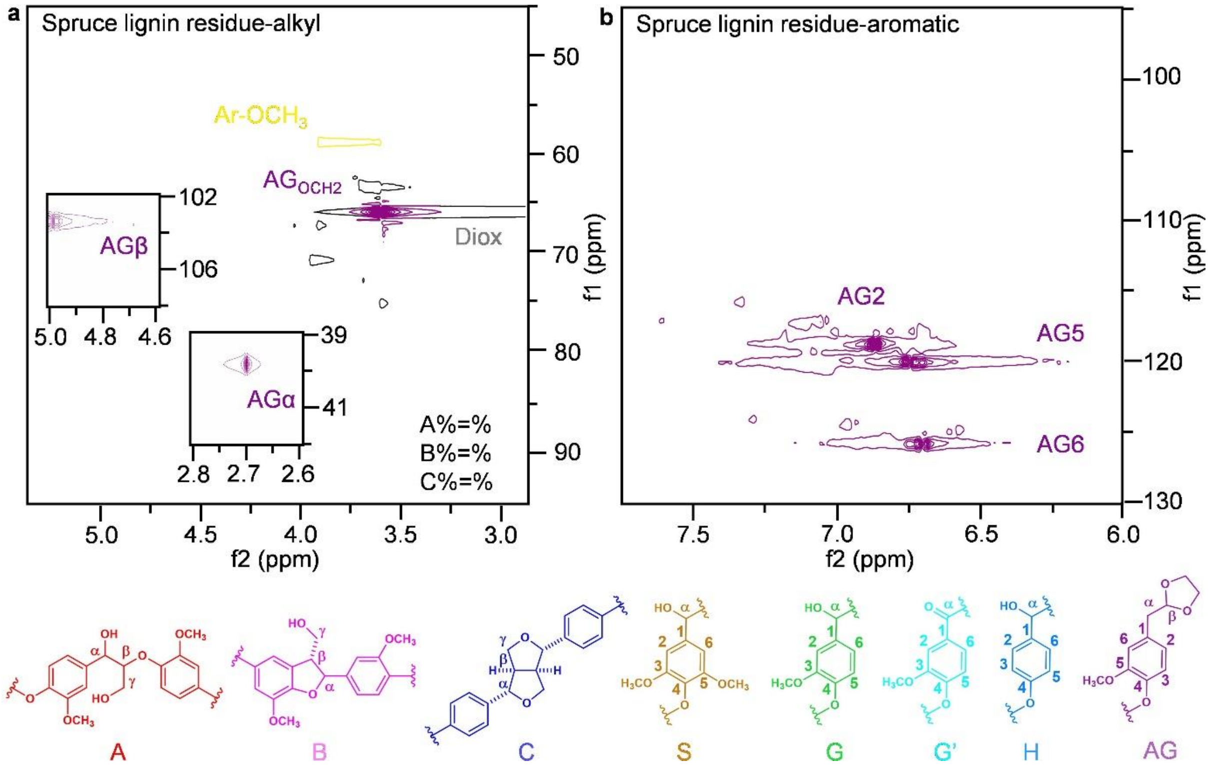

Supplementary Figure 11. (a) The alkyl region and (b) the aromatic region of the 2D-HSQC NMR spectra of the residue obtained from the acid-catalytic depolymerization of spruce lignin. Some representative structures are shown underneath.

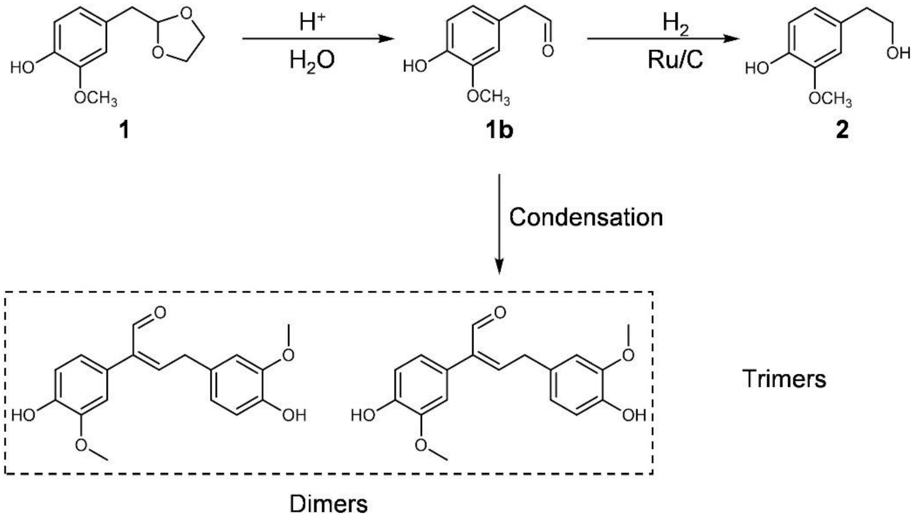

Supplementary Figure 12. Reaction path and mechanism for deprotection of 1 into 2.

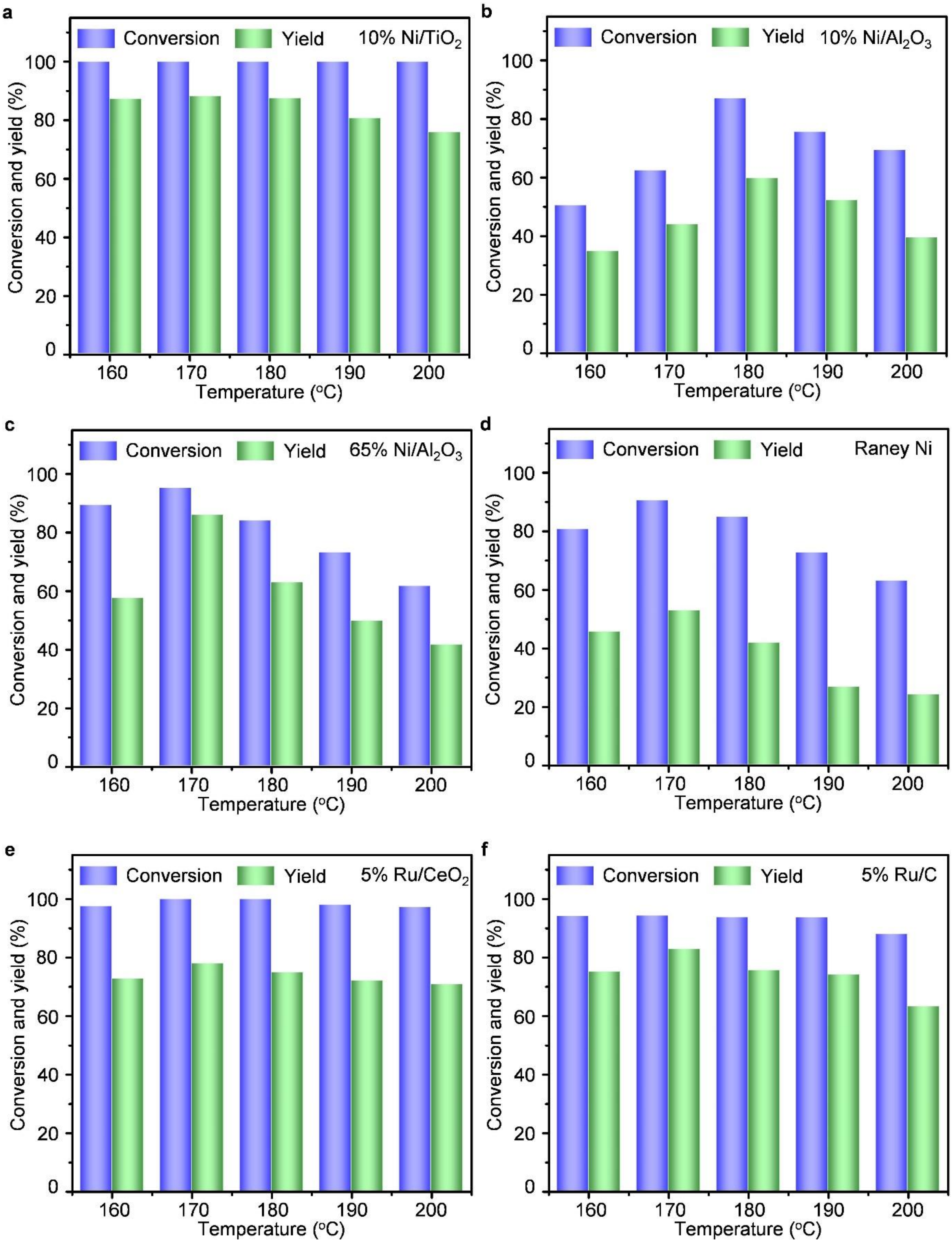

Supplementary Figure 13. Establishing optimal reaction conditions for the catalytic conversion of 2 to 3. Catalytic results for the hydrogen-borrowing amination of a **2** in a fix-bed reactor over different catalysts. Reaction condition: catalyst (0.5g), **2** in *p*-xylene solution (0.025 M), NH_3 pressure (0.6 MPa), liquid flow rate (0.3 mL min^{-1}), gas flow rate (100 mL min^{-1}), WHSV (0.163 h^{-1}).

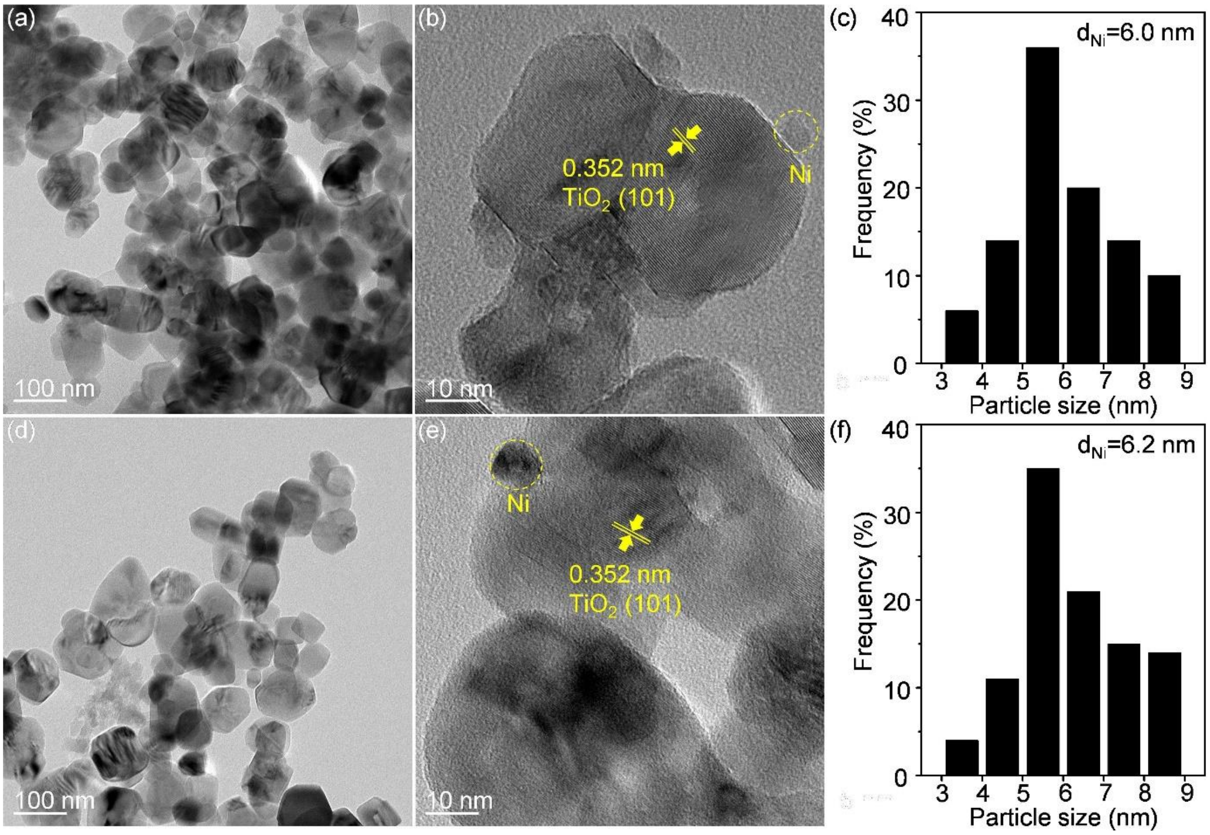

Supplementary Figure 14. TEM images and Ni particle size distribution of catalysts. Representative TEM images and Ni particle size distribution of the fresh (**a, b, c**) and used 10%Ni/TiO_2 (**d, e, f**).

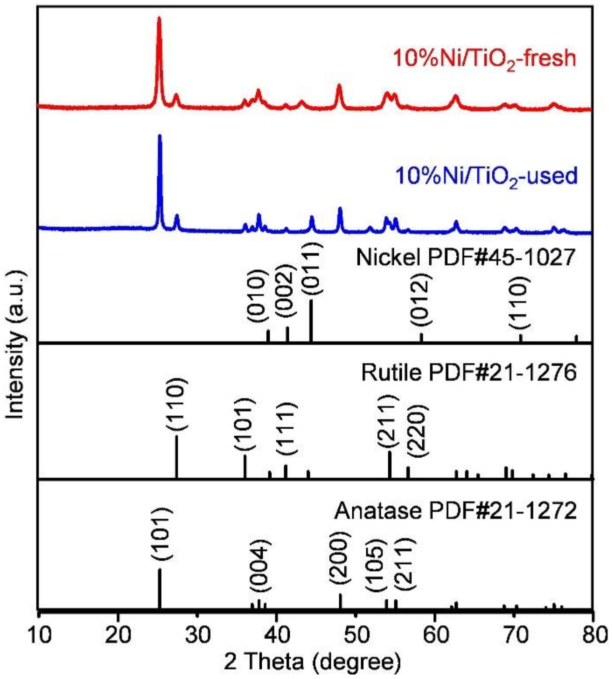

Supplementary Figure 15. XRD patterns of the fresh and used 10%Ni/TiO_2.

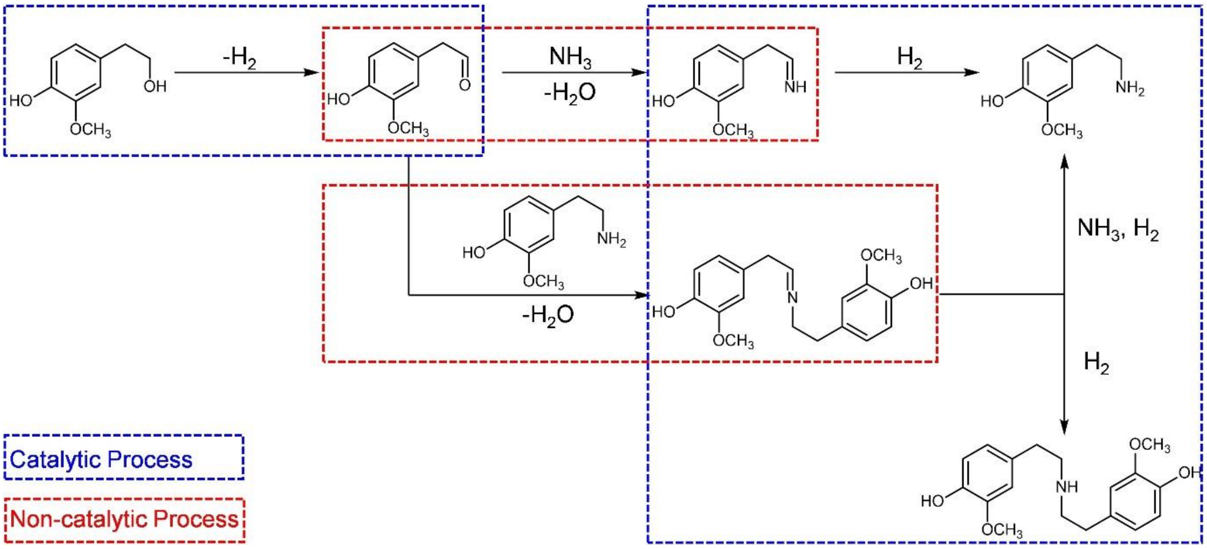

Supplementary Figure 16. The reaction network in hydrogen-borrowing amination of 2.

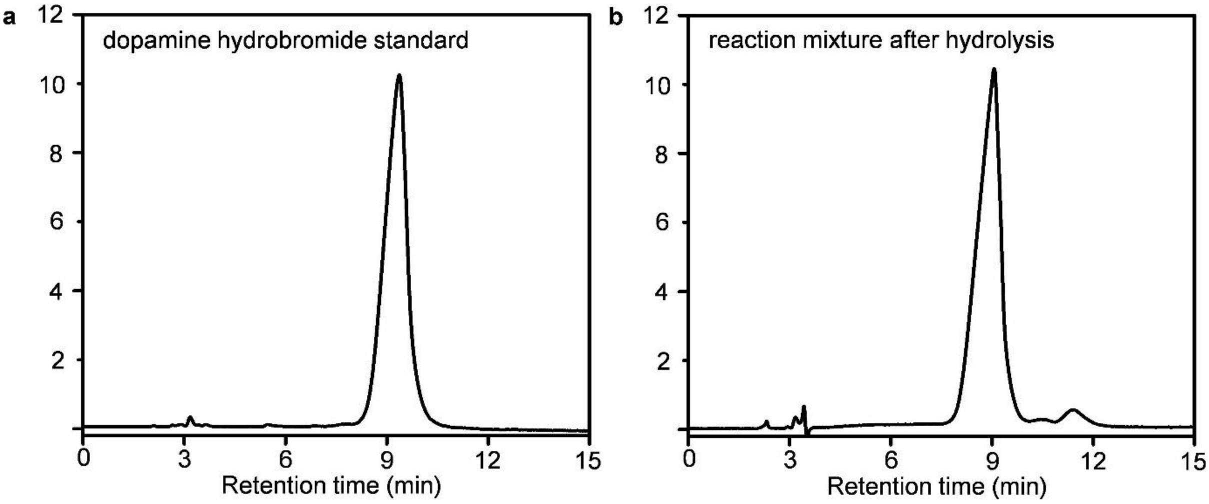

Supplementary Figure 17. Representative HPLC chromatographs of dopamine hydrobromide standard and reaction mixture after hydrolysis of 3 recorded at 280 nm. Reaction condition: **3** (1.0 g), HBr (6.0 g), N_2 pressure (0.1 MPa), temperature (120 °C), 6h.

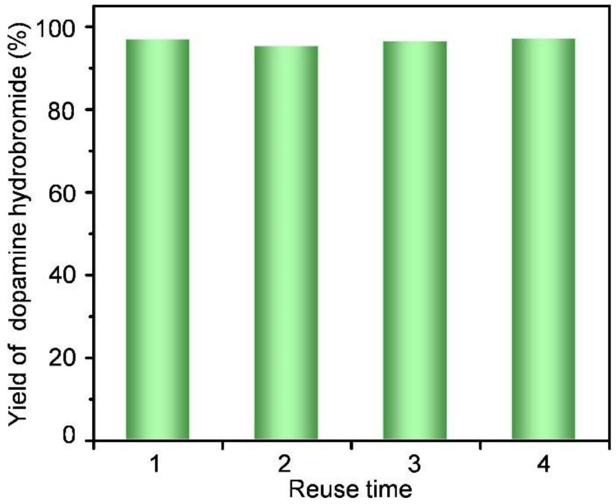

Supplementary Figure 18. Stability test of the hydrolysis of 3 to 4a. Performance of the HBr catalyst in four consecutive tests with reuse of the catalysts. Reaction condition: **3** (1.0 g), acid (6.0 g), N_2 pressure (0.1 MPa), temperature (120 ℃), 6h.

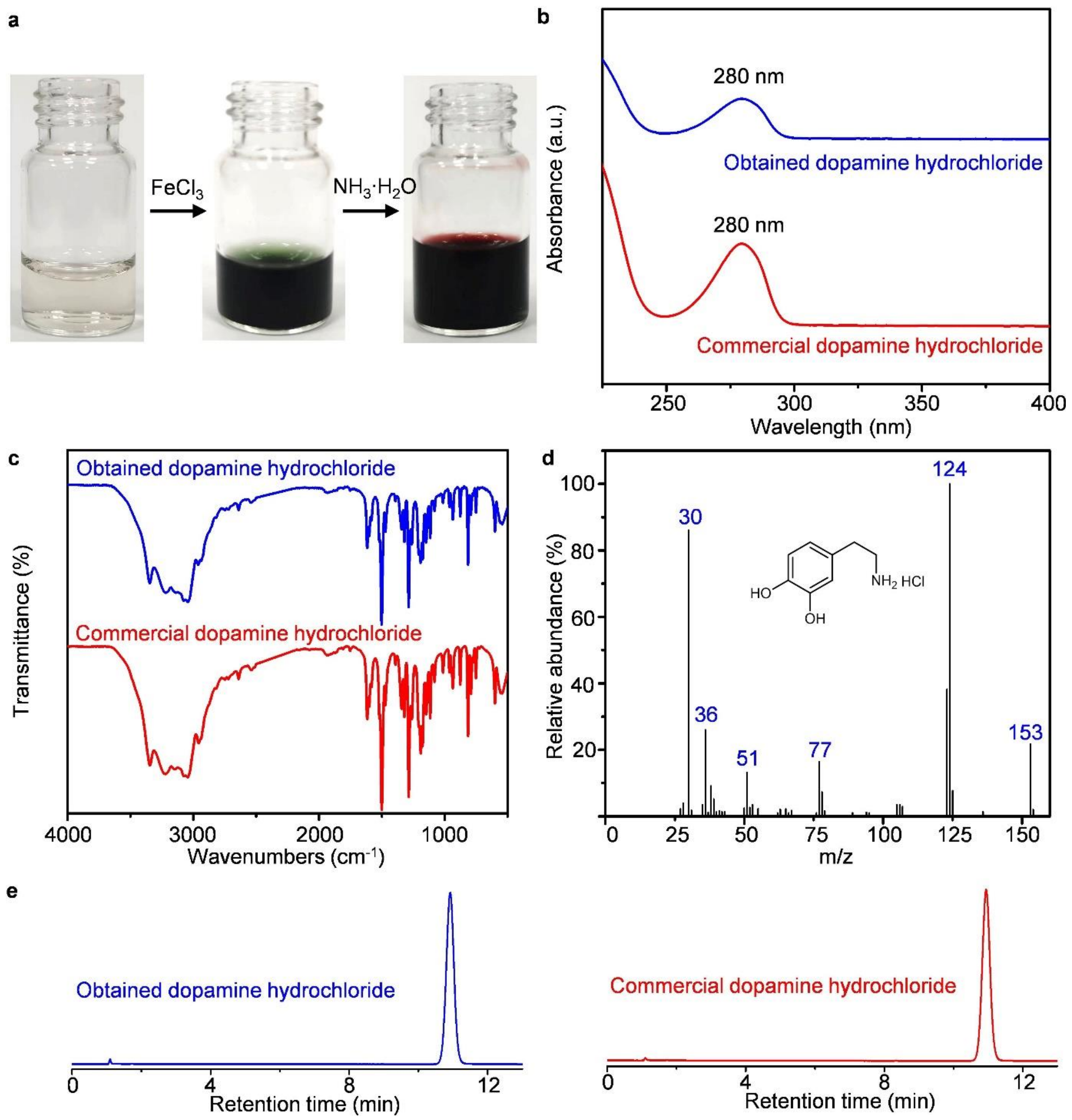

New Supplementary Figure 19. The qualitative and quantitative analysis of dopamine hydrochloride obtained from dopamine hydrobromide in ethanol. a Pictures for the color test of obtained dopamine hydrochloride aqueous solution. **b** The UV-Vis spectrums of obtained and commercial dopamine hydrochloride. **c** The FT-IR spectrums of obtained and commercial dopamine hydrochloride. **d** The mass spectrum of obtained dopamine hydrochloride. **e** Representative HPLC chromatographs of obtained and commercial dopamine hydrochloride recorded at 280 nm.

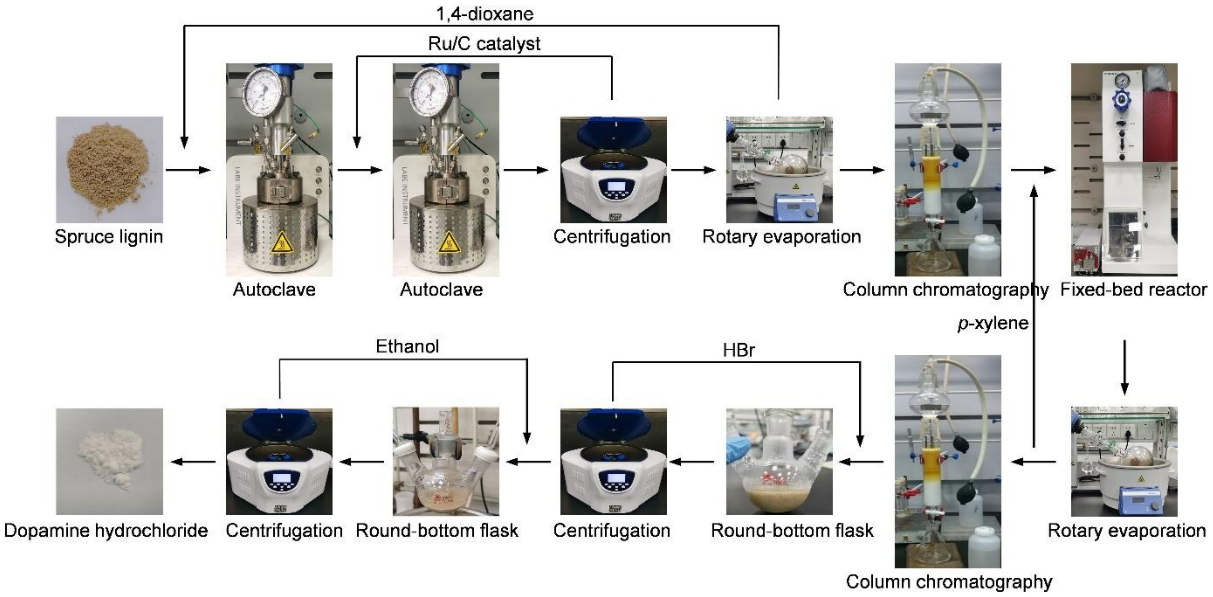

Supplementary Figure 20. Photos and process yield for the production of dopamine hydrochloride from spruce lignin as the sole substrate.

Supplementary Tables

Supplementary Table 1. The content of major linkages and the subunits ratio of different lignin.

Softwood lignin	Ratio of major linkages (%)			Ratio of subunits (%)			Theoretical yield of dopamine hydrochloride (wt.%)
	β-O-4	*α*-O-4	*β*-*β*	S	G	H	
Spruce	68%	28%	4%	0%	98%	2%	9.8
Pine	62%	32%	6%	1%	96%	3%	7.9
Cedar	53%	24%	23%	0%	98%	2%	4.8
Douglas fir	64%	30%	6%	1%	97%	2%	8.5

[a]The data were obtained *via* 2D-HSQC NMR characterization.
S refers to syringyl units, G refers to guaiacyl units, H refers to *p*-hydroxyphenyl units.

Supplementary Table 2. Catalyst and solvent screening for 1 production in lignin acidolysis with ethylene glycol stabilization.[a]

Entry	Lignin	Catalyst	Solvent	Yield of **1** (wt. %)	Depolymerization efficiency (%)[b]
1	Spruce	H_2SO_4	1,4-dioxane	10.3	95.4
2	Spruce	H_2SO_4	toluene	1.4	13.0
3	Spruce	H_2SO_4	methanol	3.6	33.3
4	Spruce	H_2SO_4	dimethyl carbonate	6.4	59.3
5	Spruce	TfOH	1,4-dioxane	10.1	93.5
6	Spruce	HCl	1,4-dioxane	0	0
7	Spruce	HNO_3	1,4-dioxane	0	0
8	Spruce	HF	1,4-dioxane	0	0

[a]Reaction conditions: spruce lignin (0.2 g), ethylene glycol (0.72 mL), solvent (30 mL), acid (16 μL), temperature (140 °C), 1 h.
[b]Depolymerization efficiency (%) = yield of **1** / theoretical yield of **1**. The theoretical yield of **1** in spruce lignin was 10.8 wt.%, obtained by combining the 2D-HSQC NMR method and derivatization followed by the reductive cleavage method.

Supplementary Table 3. The GC-FID chromatogram of lignin oil obtained after spruce lignin depolymerization.

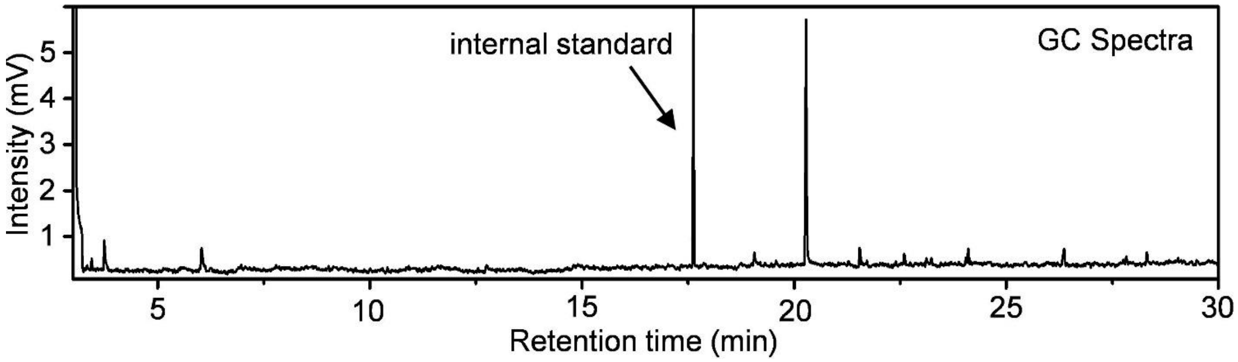

Entry	Ret. (min)	Compound	Yield (wt.%)	Entry	Ret. (min)	Compound	Yield (wt.%)
1	3.035	1,4-dioxane	--	6	21.555	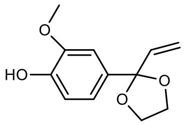	0.5
2	3.740	Ethylene glycol	--	7	22.600	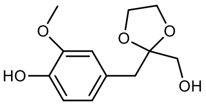	0.2
3	17.061	Pentadecane	--	8	24.105	Dimers	0.3
4	19.061	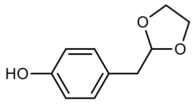	0.3	9	26.365	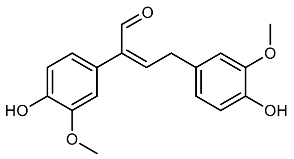	0.3
5	20.280	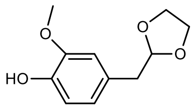	10.3	10	28.315	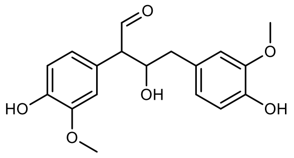	0.2

Reaction conditions: spruce lignin (0.2 g), ethylene glycol (0.72 mL), 1,4-dioxane (30 mL), H_2SO_4 (16 µL), temperature (140 °C), 1 h.

Supplementary Table 4. Results for deprotection of 1 into 2.

Entry	Reaction condition				Yield (%)			Conv. (%)
	Ru/C (g)	H_2/N_2 (MPa)	H_2O (mL)	Time (h)	**1b**	**2a**	**2**	
1	0.1	1	--	1	0.7	11.5	23.3	43.1
2	--	--	1	1	77.5	0	0	80.9
3	--	--	1	6	7.3	0	0	100
4	0.1	1	1	1	0	0	90.3	99.7
5	0.1	1	1	6	0	0	90.1	100

Reaction conditions: **1** (0.02 g), 1,4-dioxane (30 mL), H_2SO_4 (16 uL), 120 °C.

Supplementary Table 5. Summary of physical properties of the fresh and used 10%Ni/TiO_2 catalysts.

Entry	Catalyst	Surface area (m^2 g^{-1})	Pore volume (cm^3 g^{-1})	Pore size (nm)	Ni content[a] (wt.%)
1	10%Ni/TiO_2-fresh	48	0.296	3.7	8.5
2	10%Ni/TiO_2-used	44	0.284	3.5	8.2

[a]Determined by ICP-AES.

Supplementary Table 6. Assumptions for equipment cost.

Item description	Equipment cost (CNY)	Equipment cost and other direct costs (CNY)
pump 1	395200	707200
Reactor 1	11719900	14831600
Cooler 1	53700	649100
Makeup H_2/N_2	5565400	7017100
Pump 2	257300	418100
Reactor 2	11913100	15037300
Tower 1	1134900	3154000
Makeup NH_3	2167600	2830500
Pump 3	271300	446500
Reactor 3	599800	1494063
Tower 2	1134900	3154000
Pump 4	255900	401200
Reactor 4	749400	1904600
Separation 1	1046900	1722700
Pump 5	255500	395800
Reactor 5	1001600	2194900
Separation 2	1046900	1722700
total	39569300	58081363

Supplementary Table 7. Assumptions for electricity cost of equipment.

Item description	Power (KW)	Electricity (KW·h)	Total cost (CNY)
pump 1	45	360000	180000
Reactor 1	150	1200000	600000
Cooler 1	3	24000	12000
Makeup H_2/N_2	190	1520000	760000
Pump 2	3	24000	12000
Reactor 2	160	1280000	640000
Tower 1	--	--	--
Makeup NH_3	1.11	8880	4440
Pump 3	7.5	60000	30000
Reactor 3	--	--	--
Tower 2	--	--	--
Pump 4	1.5	12000	6000
Reactor 4	22.7	181600	90800
Separation 1	15	120000	60000
Pump 5	1.5	12000	6000
Reactor 5	18.5	148000	74000
Separation 2	15	120000	60000
Total		5070480	2535240

Supplementary Table 8. Assumptions for raw material cost.

Items	Unit price (CNY/t)	Quantity(t/year)	Total cost (CNY/year)
Lignin	1000	10000	10000000
Sulfuric acid	514.9851	1464.4	754144.1804
Ethylene glycol	8123.2934	234.9	1908161.62
1,4-dioxane	14900	15703	233974700
process water	15	50000	750000
N_2	795.6	3500	2784600
H_2	10000	500	5000000
Ru/C (5 wt.%)	3260400	250	815100000
Paraxylene	6902.2281	1546.3	10672915.31
NH_3	2438.8256	75.89	185082.4748
Ni/TiO_2 (10wt%)	121857	1250	152321250
HBr	16000	59.13	946080
Ethanol	5000	55	275000
HCl	5000	125	625000
Total			1235296934

Supplementary Table 9. Assumptions for the estimation of the total product cost

Component		Base		Cost (CNY)
Equipment cost and other direct costs	1.1	Installed cost of all equipment		58081363
Indirect costs	1.2	60% of 1.1		34848817.8
Raw materials	2.1	All costs of raw materials and catalysts		1235296934
Utilities	2.2	Cooling water	3 CNY/t	820800
		Electricity	0.5 CNY/kW·h	2535240
Operating and maintenance cost	2.3.1	Operating labors	9 operators, 100000 CNY/operator/year	900000
	2.3.2	Direct supervisory and clerical labor	20% of (2.3.1)	180000
	2.3.3	Maintenance and repairs	10% of fixed capital	9293018.1
	2.3.4	Operating supplies	2% of fixed capital	1858603.6
	2.3.5	Laboratory charge	15% of (2.3.1)	135000
Depreciation	2.4	Straight line depreciation, life period 20y, salvage value 4%		4460648.7
Plant overhead cost	2.5	60% of 2.3.1+2.3.2+2.3.3		6223810.9
Administrative cost	2.6	2% of the product cost		27092684.7
Distribution and selling cost	2.7	2% of the product cost		27092684.7
Unit price of dopamine hydrochloride (CNY/t)				2201280.6

Supplementary References

1. Lancefield, C. S., Ojo, O. S., Tran, F. & Westwood, N. J. Isolation of functionalized phenolic monomers through selective oxidation and C-O bond cleavage of the *β*-O-4 linkages in lignin. *Angew. Chem. Int. Ed.* **54**, 258-262 (2015).
2. Deuss, P. J. *et al.* Aromatic monomers by in situ conversion of reactive intermediates in the acid-catalyzed depolymerization of lignin. *J. Am. Chem. Soc.* **137**, 7456-7467 (2015).
3. Lu, F. & Ralph, J. DFRC method for lignin analysis. 1. new method for *β*-aryl ether cleavage: lignin model studies. *J. Agric. Food Chem.* **45**, 4655-4660 (1997).
4. De Santi, A., Galkin, M. V., Lahive, C. W., Deuss, P. J. & Barta, K. Lignin-first fractionation of softwood lignocellulose using a mild dimethyl carbonate and ethylene glycol organosolv process. *ChemSusChem* **13**, 4468-4477 (2020).
5. Kohno, M., Sasao, S. & Murahashi, S-I. Synthesis of phenethylamines by hydrogenation of β-nitrostyrenes. *Bull. Chem. Soc. Jpn.* **63**, 1252-1254 (1990).
6. Wang, Y., Fice, D., S. & Yeung, P. A simple high-performance liquid chromatography assay for simultaneous determination of plasma norepinephrine, epinephrine, dopamine and 3,4-dihydroxyphenyl acetic acid. *J. Pharm. Biomed. Anal.* **21**, 519-525 (1999).
7. Bernini, R., Crisante, F., Barontini, M. & Fabrizi, G. A new and efficient route for the synthesis of naturally occurring catecholamines. *Synthesis*, **22**, 3838-3842 (2009).
8. Velcicky, J., Soicke, A., Schmalz, H-G. & Murahashi, S-I. Palladium-catalyzed cyanomethylation of aryl halides through domino suzuki coupling-isoxazole fragmentation. *J. Am. Chem. Soc.* **133**, 6948-6951 (2011).
9. Cognitive Market Research. *Global dopaimne market report 2022*. https://www.cognitivemarketresearch.com/dopamine-market-report
10. Liao, Y. H. *et al.* A sustainable wood biorefinery for low-carbon footprint chemicals production. *Science* **367**, 1385-1390 (2020).
11. Fang, Q., Jiang, Z., Guo, K., Liu, X., Li, Z., Li, G. & Hu, C. Low temperature catlytic conversion of oligomers derived from lignin in pubescens on Pd/$NbOPO_4$. *Appl. Catal. B: Environ.* **263**, 118325 (2020).
12. Upton, B. M. & Kasko, A. M. Strategies for the conversion of lignin to high-value polymeric materials: review and perspective. *Chem. Rev.* **116**, 2275-2306 (2016).

13. Franco, R., Reyes-Resina, I. & Navarro, G. Dopamine in health and disease: much more than a neurotransmitter. *Biomedicines* **9**, 109 (2021).

14. Berke, J. D. What does dopamine mean? *Nat. Neurosci.* **21**, 787-793 (2018).

15. Piggott, M. A. *et al.* Striatal dopaminergic markers in dementia with Lewy bodies, Alzheimer's and Parkinson's diseases: rostrocaudal distribution. *Brain* **122**, 1449-1468 (1999).

16. Johnson, K. A. *et al.* Combined dopamine transporter and FDG PET in dementia with Lewy bodies, Alzheimer's disease, and Parkinson's disease. *Neurology* **62**, A300-A300 (2004).

17. Zhang, A., Neumeyer, J. L. & Baldessarini, R. J. Recent progress in development of dopamine receptor subtype-selective agents: potential therapeutics for neurological and psychiatric disorders. *Chem. Rev.* **107**, 274-302 (2007).

18. Lee, H. A., Ma, Y., Zhou, F., Hong, S. & Lee, H. Material-independent surface chemistry beyond polydopamine coating. *Acc. Chem. Res.* **52**, 704-713 (2019).

19. Mei, S., Xu, X., Priestley, R. D. & Lu, Y. Polydopamine-based nanoreactors: synthesis and applications in bioscience and energy materials. *Chem. Sci.* **11,** 12269-12281 (2020).

20. Natte, K., Narani, A., Goyal, V., Sarki, N. & Jagadeesh, R. V. Synthesis of functional chemicals from lignin-derived monomers by selective organic transformations. *Adv. Synth. Catal.* **362**, 5143-5169 (2020).

21. Chen, Z. W., Zeng, H. Y., Gong, H., Wang, H. N. & Li, C-J. Palladium-catalyzed reductive coupling of phenols with anilines and amines: efficient conversion of phenolic lignin model monomers and analogues to cyclohexylamines. *Chem. Sci.* **6**, 4174-4178 (2015).

22. Pelckmans, M., Renders, T., Van de Vyver, S. & Sels, B. F. Bio-based amines through sustainable heterogeneous catalysis. *Green Chem.* **19**, 5303-5331 (2017).

23. Sun, Z. H. *et al.* Complete lignocellulose conversion with integrated catalyst recycling yielding valuable aromatics and fuels. *Nat. Catal.* **1**, 82-92 (2018).
